# A Rule-Based Conversational Agent for Mental Health and Well-Being in Young People: Formative Case Series During the Rise of Generative AI

**DOI:** 10.2196/69841

**Published:** 2025-12-01

**Authors:** Aimee-Rose Wrightson-Hester, Gee Anderson, Joel Dunstan, Peter M McEvoy, Christopher J Sutton, Bronwyn Myers, Sarah J Egan, Sara Jane Tai, Melanie Johnston-Hollitt, Wai Chen, Tom Gedeon, Joanna C Moullin, Warren Mansell

**Affiliations:** 1 Curtin enAble Institute, School of Population Health Faculty of Health Sciences Curtin University Perth, Western Australia Australia; 2 Curtin Institute for Data Science Curtin University Perth, Western Australia Australia; 3 Centre for Clinical Interventions, North Metropolitan Health Service Nedlands Australia; 4 Lancashire Clinical Trials Unit, University of Lancashire Lancaster United Kingdom; 5 Substance Use and Tobacco Research Unit South African Medical Research Council Tygerberg, Western Cape South Africa; 6 School of Health Sciences; Division of Psychology and Mental Health Faculty of Medical and Health Sciences University of Manchester Manchester United Kingdom; 7 School of Electrical Engineering, Computing and Mathematical Sciences Curtin University Perth, Western Australia Australia

**Keywords:** mental health, conversational agents, chatbots, acceptability, feasibility, co-design, artificial therapist, youth, child, acceptability, Manage Your Life Online

## Abstract

**Background:**

There is a shortage of services available to address the growing demand for mental health support in Australia and worldwide. Digital interventions, including conversational agents, can overcome barriers to accessing mental health support. The recent advances in large language models have led to an improvement in the perceived human-like naturalness of chatbot conversations, but there is little research on the experience of chatbots to support mental health. Manage your life online (MYLO) is a rule-based chatbot that was co-designed with young people and uses questions to help users explore their problems. In a case series conducted before the release of ChatGPT (OpenAI), users rated a new smartphone interface for MYLO as acceptable, and there was a large effect size for reduction in problem-related distress.

**Objective:**

This study aimed to evaluate an improved version of MYLO and compare the user experience of MYLO in this case series to the previous version that was completed in November 2022.

**Methods:**

We replicated and extended the previous 2-week case-series, conducted from September to November 2022, by testing 4-week usage of MYLO with a larger sample between October and December 2023. We recruited 24 young people living in Western Australia who self-described as having a lived experience of anxiety or depression. Participants had access to and used MYLO over a 4-week period while completing online weekly surveys that included a range of health and psychological questionnaires. After the 4-week testing phase, participants were invited to provide feedback on their experience of using MYLO through an interview or focus group discussion.

**Results:**

In total, 13 of the 24 participants were retained throughout the study and took part in interviews. On average, participants had around 4 conversations with MYLO. They experienced both benefits and limitations of these conversations. They spoke about their recent experiences with ChatGPT (released in November 2022 after the previous case-series concluded) and other generative artificial intelligence (AI) tools, stating that they had expected MYLO to possess similar functionality, which it did not. Nonetheless, we found moderate to large effect sizes for improvements in problem-related distress (Cohen *d*=–1.07), anxiety (Cohen *d*=–0.41), and psychiatric impairment (Cohen *d*=–0.60) and some evidence of reliable improvement in clinical outcomes.

**Conclusions:**

These findings have implications for mental health chatbots in the age of ChatGPT and highlight a need for researchers to engage with new technologies to improve user experience, while maintaining the necessary safety and ethical standards that can be a significant challenge for generative AI.

## Introduction

To address the growing need for mental health care [1], researchers and policymakers are investigating the use of digital and artificial intelligence (AI) solutions to improve the scalability of interventions [2]. Digital interventions might be particularly helpful for young people who have grown up with the internet and who experience barriers to accessing appropriate mental health care [3]. Conversational agents or chatbots are one technology that people are using to support their mental health. Mental health conversational agents are specifically designed to support users’ mental health by emulating various forms of traditional psychotherapy [4]. Studies have found high user satisfaction with chatbots [5,6], with users enjoying the interactive approach and building a relationship with the chatbots like that of a human therapist or friend [7,8].

Although feedback is generally positive, many studies report negative feedback from users about mental health chatbots, such as repetitive content, not feeling understood [9], inappropriate responses from the bot [7], and technical issues [10]. Before 2022, the majority of mental health chatbots were rule-based systems in that humans explicitly programmed the way that the chatbot recognized the content of the user’s text and the rules that decided how the chatbot replied using human-authored statements. Yet, in late 2022, highly naturalistic conversations with chatbots became widely accessible with the release of generative AI agents such as ChatGPT [11]. These systems use large language models to extract layers of regularities within vast databases of text, which leads to human-like responses to users that are not preprogramed or open to the direct oversight of human operators; we return to the issues this brings in the Discussion. Since ChatGPT’s release, the use of AI technology has seen substantial growth driven by improvements in natural language processing and the integration of AI technologies into day-to-day personal and business applications [12]. Our paper presents formative data on how the rise in generative AI chatbots may have impacted an existing rule-based chatbot, with recommendations for developers in this fast-paced arena.

There is increasing awareness that digital innovations, such as social media and generative AI chatbots, have the potential for psychological harm [13]. When analyzing risk cases, the harm tends to relate to the “sycophantic” provision of information or advice that confirms a user’s pre-existing beliefs [14]. Whilst providing information and advice is often beneficial in the digital domain, this is not universally the case. Indeed, misinformation has been identified as one of the greatest threats of AI [15]. Crucially, the provision of advice and information is not the main ingredient of effective psychological therapy and counselling. Core ingredients of effective therapy include nonjudgmental communication, active listening, and curious inquiry [16]. Yet, the conversational style of most existing chatbots, whether or not they target mental health or use generative AI systems, tends to focus on the provision of advice, interpretation, and information instead of these active therapeutic ingredients. This leaves a gap for innovation for a chatbot that does not provide advice, potential misinformation, or echo-chamber amplification. As such, MYLO is unique because it specializes in asking curious questions to help the user explore a problem in depth and detail, and notice current thoughts and feelings about the problem [17].

Manage your life online (MYLO) is a conversational agent that uses rule-based AI to exchange messages with the user. MYLO aims to emulate a conversation like that of a method of levels therapist [18]. Method of levels therapists help clients explore internal conflicts between their current state and their ideal state [19]. It is these conflicts that lead to psychological distress according to perceptual control theory, which underpins the method of levels therapy [6,18-20]. MYLO responds to user input in real time with curious questions tailored to the user’s current problem. These questions aim to help the user sustain their awareness of the conflicts they are experiencing and explore the root cause of their distress [21].

**Figure 1:**
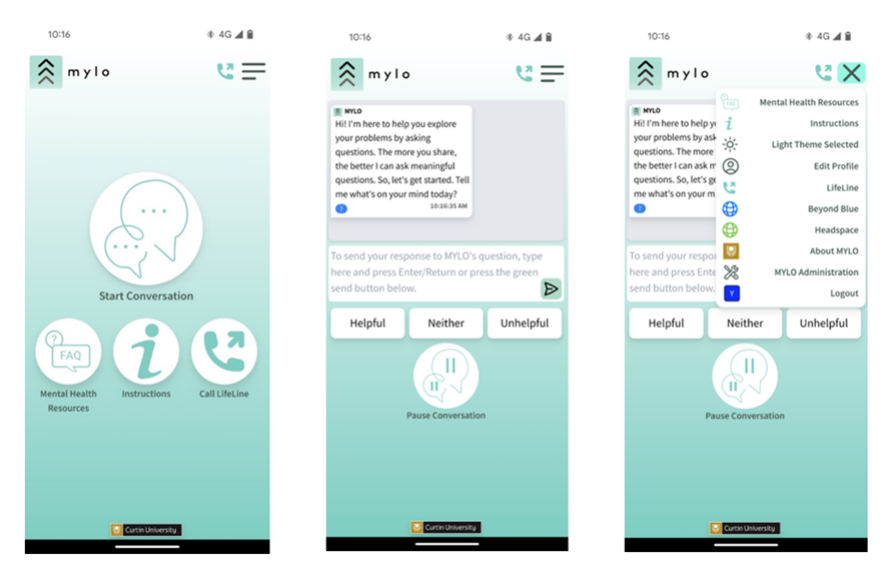
Screenshots of the MYLO Interface showing, from left to right, the home screen, the start to a conversation, and the drop-down menu. MYLO: Manage your life online.

MYLO was initially developed for use on a desktop, and evaluated when used for short sessions in controlled trials within an experimental context [21]. The current MYLO interface (refer to Figure 1) was co-designed with young people as a progressive web application on a smartphone to be accessible and appealing to use [22]. The acceptability of the current interface was evaluated by young people in a case-series in which users tested MYLO for 2 weeks and answered weekly online surveys [22]. Participants rated MYLO as acceptable on the system usability scale (mean 73.57, SD 16.02) and reported that the interface was easy to use and that they liked its functionality in interviews and focus groups. In total, 6 of the 8 participants who attended an interview or focus group said they would recommend MYLO to their friends. Further, reliable change scores [23] were calculated for each participant who completed baseline and posttesting (n=11), and 7 participants showed reliable improvements in their problem-related distress or other clinical outcome (such as depression, anxiety, and psychiatric impairment) scores. Due to the modest sample size and short testing phase (2 weeks), no formal statistical testing was performed.

Following the previous case series, we made essential changes that we deemed necessary to enhance MYLO’s conversational ability and establish safety before moving toward a full-scale trial. We used this case series to assess the acceptability and feasibility of these changes. We updated the MYLO database based on user feedback, alongside guidance from a new youth advisory panel. Specifically, new questions, themes, and terms were added to assist users with a wider range of problems. Importantly, 2 themes to identify risk of suicide or nonsuicidal self-harm were added, which, when triggered, responded to users by reminding them of the local mental health crisis hotline accessible through the MYLO interface. Relatedly, we added a measure of events to assess safety ahead of a full trial. During this development phase, the ability to decrypt user conversations was also added. Analyzing user conversations will allow researchers to identify the helpful and unhelpful elements of MYLO’s conversations, building on the work of Gaffney et al [21]. While members of the youth advisory panel had shared sections of their conversations to discuss with the research team, it was unclear whether research participants would consent to share their conversations during a research trial.

In this study, we, therefore, aimed to extend the previous case-series by testing MYLO for a longer period of time and to test the changes made to the methodology described above before conducting a full-scale randomized controlled trial.

We also assessed the feasibility of conversation decryption and our methodology for gaining consent for this procedure. We did not have a target number of conversations or participants. We report the method used and then the number of participants and conversations obtained. We explored the user experience of MYLO using the session impact scale, the system usability scale, and qualitative interviews and focus groups. We also evaluated the effect size with regard to changes in problem-related distress, anxiety, depression, psychiatric impairment, and self-reported ability to engage in goal conflict reorganization after 4 weeks of using MYLO. We compared all of our findings to the previous case series, and through the lens of the widespread rise in use of generative AI chatbots that occurred in the year between the 2 case series. This part of the study is naturalistic, given the timing of the study, rather than making a direct comparison between generative AI and rule-based chatbots.

## Methods

### Design

The case-series protocol was similar to Wrightson-Hester et al [22], which was conducted from September to November 2022 during which participants engaged in the intervention over a period of 2 weeks. In the current study, the intervention duration was extended from 2 to 4 weeks, between October and December 2023. Therefore, a brief method will be described here, but for full details, see Wrightson-Hester et al [22]. One major difference was the procedure used to gain consent for the decryption and analysis of conversations, which will be described in full in the procedure. To measure any adverse impacts of using MYLO, we also added an adverse events survey post testing.

### Ethical Considerations

Approval for the research was obtained from the Curtin University Human Research Ethics Committee (HREC2022-0466). All participants were presented with an information sheet describing the aims and procedures of the study as well as information regarding how their data would be used, and they indicated their consent for each aspect of the study. A subject-generated code was used to link data across phases of the study anonymously. Our recruitment of 16- to 17-year-olds followed advice from ethical guidelines and our consumer panel, and involved either voluntary parental consent or a competency assessment to establish that the participant could be considered a mature minor. Participants were reimbursed for their time completing the online surveys, as well as for the focus group or interview with a digital gift card at a rate of Aus $20 (US $13, A currency exchange rate of Aus $1=US $0.65 is applicable) per hour with a maximum commitment of 5 hours if all surveys and an interview or focus group was completed (total of Aus $100 [US $65]). This report has been prepared so that the identification of individual participants is not possible.

### Participants

The study was advertised on social media via Metaverse, X (X Corp, formerly known as Twitter), and LinkedIn (Microsoft) and shared by members of the research group through their existing networks and personal social media pages. The inclusion criteria were (1) being aged 16 to 24 years, (2) currently living in Western Australia, (3) self-reported an experience of anxiety or depression (either current or past), (4) having access to a smartphone and the internet, and (5) being able to read and type in English. The exclusion criteria were (1) scoring greater than 20 on the PHQ-9 (Patient Health Questionnaire-9), the threshold for severe depressive symptoms, or (2) reporting the experience of frequent suicidal thoughts (by scoring 2 or more on the PHQ-9 item 9) [24]. To partly address issues of inclusivity and generalizability, we ensured a minimum of 2 participants of each of the following: male, female, nonbinary gender, and non-Australian living in regional or remote Western Australia. Participants expressed their interest in participating through an online survey via Qualtrics (Qualtrics International Inc) and completed questions to assess their eligibility and demographic information, and gave informed consent to participate along with their contact details.

There was no formal sample size calculation as the purpose of the trial was not to test for effectiveness of MYLO. The initial sample size of 24 was deemed sufficient to assess usability and acceptability of MYLO, and, in particular, we aimed for a larger sample size than the previous case series, to ensure that, after attrition, we had a similar number as the last case series in order to make comparisons.

### Materials

#### Web-Based Survey

Participants completed anonymous online surveys hosted by Qualtrics at baseline, then after each week of testing for a total of 4 weeks, between October and December 2023. A subject-generated identification code [25] was used to link participants’ surveys across time points. The self-report questionnaires included in the online survey are presented in Table 1 (this table is adapted from Wrightson-Hester et al [22]).

**Table 1:** The names and properties of the self-report questionnaires.

Questionnaire	Measures	Scoring
Patient Health Questionnaire-9 [24]	9 items; depression	0-4: minimal depression, 5-9: mild depression, 10-14: moderate depression, 15-19: moderately severe depression, and 20-27: severe depression.
Generalized anxiety disorder assessment-7 [26]	7 items; anxiety	0-4: minimal anxiety, 5-9: mild anxiety, 10-14: moderate anxiety, and 15-21: severe anxiety.
General health questionnaire-12 [27]	12 items; psychiatric impairment	Traditional (acute) scoring method used. Scores range from 0 to 12, and higher scores indicate a greater possibility of psychological distress.
Short Form-6D version 2 [28]	6 items; general health	Scores range from −0.685 to 1, with 1 indicating perfect health. Australian weights were used for this sample.
Psychological outcome profiles [29]	4 items used for scoring; change in problem-related distress over the course of therapy	Scores range from 0 to 20. Decreases in scores between pretherapy and posttherapy indicate that a positive change has occurred.
Reorganization of conflict scale [30]	10-item subscale; goal conflict awareness and the proposed mechanism of change in the method of levels therapy	Each item is scored from 0 (I do not believe this at all) to 100 (I believe this completely). The mean of the 10 items is used as the outcome.
General self-efficacy scale [31]	10 items; self-efficacy	Scores range from 10 to 40. Higher scores indicate higher perceived general self-efficacy.
Session impact scale^a^ [32]	17 items; session (therapeutic) satisfaction	Each item is scored from 1 (not at all) to 5 (very much). We calculated the mean scores for the unwanted thoughts, relationship impacts, hindering impacts, understanding, and problem-solving subscales. Item 17 measures “other impacts,” an optional item that is not used in scoring.
System usability scale^a^ [33]	10 items; user experience of digital systems	Outcome is a percentile ranking from 0 to 100, with scores >68 considered above average.
Adverse event survey^b^	26 items; adverse events	The items have been adapted from the FOCUS clinical trial [34]. Each item is scored from 0 (Not at all) to 4 (Very much). Participants can also select “Not applicable.”

^a^Denotes questionnaires that were only used after the baseline survey.

^b^This survey was included in the posttesting (week 4) survey and also sent to noncompleters separately.

#### MYLO

Participants accessed MYLO as a progressive web application through a link provided by the research team. MYLO’s interface allows users to engage in a text-to-text conversation, access other mental health resources (including a local suicide crisis center hotline), pause and resume conversations, and alter the colors used in MYLO and their own avatars (colored squares with the user’s initial). MYLO asks users curious questions in response to free-text provided by the user, for example, if a user states they feel nervous, MYLO might ask, “What do you do when you feel nervous?” The question that MYLO presents is determined by an algorithm that picks the best question based on key terms identified in the user’s text and previous ratings of the question and term pairings. The questions aim to sustain the user’s awareness of their problems, explore them in new ways, and ultimately, resolve them through shifts in their thinking or actions to resolve them.

### Procedure

**Figure 2:**
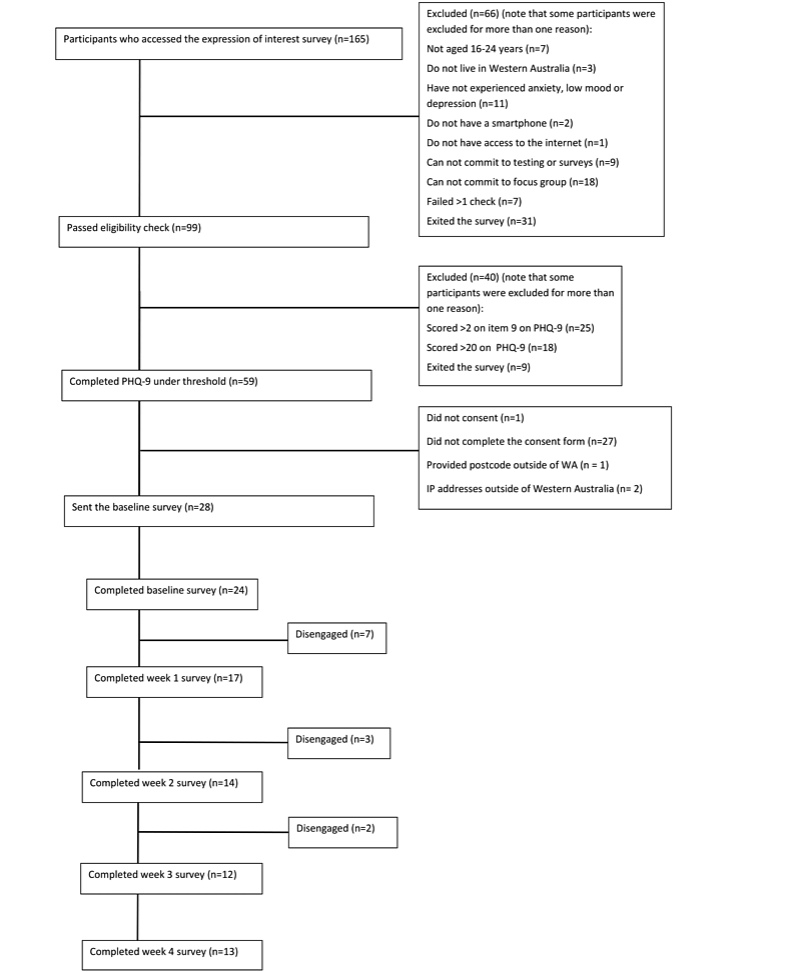
Participant flowchart through the study from Expression of Interest to posttesting survey.

Participants accessed the Expression of Interest survey via links or QR codes on study adverts. Participants completed questions to access their eligibility (refer to Figure 2), recorded their demographic information (age, gender, cultural background, sexuality, and whether or not they lived in a metropolitan versus regional, rural, or remote location), completed the PHQ-9, and provided their contact details. Eligible participants from the Expression of Interest survey were then contacted by the research team and invited to complete the baseline survey via email or text. Those who completed the baseline survey were then provided with a MYLO account (via Auth0 [Okta]) and sent the link and instructions to download MYLO. Participants then had access to and used MYLO for 4 weeks and completed weekly online surveys between October and December 2023. After the testing phase, participants were invited to attend an online focus group or an individual interview to provide further feedback on their time using MYLO. All focus groups and interviews were conducted in December 2023. The same topic guide was used to guide the interviews as was used in the previous case-series (Multimedia Appendix 1; see [22]).

At the end of the interview or focus group, participants were informed of the research team’s intent to ask for their consent to decrypt and analyze the conversations they had with MYLO during the testing phase. After the interview or focus group, case-series participants were emailed an information sheet and consent form for the decryption of their conversations. Participants were able to ask any questions, and willing participants returned their consent forms via email. Emails with the information sheet and consent form were also sent to all other participants who did not attend an interview or focus group or complete all the surveys when they were sent their gift card for taking part in the study. In the same email, noncompleters were also provided a link to complete the optional adverse events survey.

### Data Analysis

Analysis was primarily descriptive, using mean (SD), median (IQR [range]), and frequency (%), as appropriate. In addition, (standardized) effect size estimates, with 95% CIs, were computed to assess MYLO’s impact on user wellbeing and experience. We also calculated whether any participants achieved a reliable improvement or deterioration [23] during the testing phase on each of the quantitative outcome measures described previously.

To further assess users’ experience of MYLO, interviews and focus groups were conducted online via Microsoft Teams, using the same topic guide as Wrightson-Hester et al [22]. Transcription and analysis were conducted by the first author according to Vears and Gillam’s [35] inductive content analysis procedure. Coding was iterative, and emerging schemas were discussed and refined at regular research meetings between the first and last author to support confirmability. As the sample size was predefined by the case-series methodology, data saturation did not guide the number of focus groups or interviews conducted. However, the number of participants falls within the range commonly reported for data saturation.

## Results

### Participants

Figure 2 shows how many participants accessed the Expression of Interest survey and how many were lost at each stage of the survey. This process left 28 eligible participants who were contacted to participate in the study. Of these, 24 completed the baseline survey. The demographics of the participants are summarized in Table 2 and met our diversity targets. Of the 24 participants, 17 completed the week 1 survey, 14 completed week 2, 12 completed week 3, and 13 (54.2%) completed week 4. The 13th respondent in week 4 was unable to complete the week 3 survey. Focus groups and interviews were conducted with 13 participants. In total, 8 (7 completers, 1 noncompleter) participants completed and consented to the analysis of the adverse events survey.

**Table 2:** Participant demographics at baseline in the total sample and in completers.

Categories		Total sample (n=24), n (%)	Completers (n=13), n (%)
**Age (years)**			
	16-17	12 (50)	9 (69)
	18-19	5 (21)	1 (8)
	20-21	2 (8)	0 (0)
	22-24	5 (21)	3 (23)
**Gender**			
	Woman	13 (54)	8 (62)
	Man	7 (29)	3 (23)
	Nonbinary or self-described^a^	4 (17)	2 (15)
**Sexuality**			
	Heterosexual	9 (37)	3 (23)
	Non-heterosexual	11 (46)	6 (46)
	Did not disclose	4 (17)	4 (31)
**Culture**			
	Australian	14 (58)	9 (69)
	Australian and other^b^	6 (25)	3 (23)
	Other^c^	4 (17)	1 (8)
**Metropolitan or regional area**			
	Metropolitan	19 (79)	10 (77)
	Regional or Remote	5 (21)	3 (23)

^a^Three participants described themselves as nonbinary and one as a transgender male.

^b^Australian and other (self-described): Chinese, Dutch and Sri Lankan, English, Italian, Hispanic, and Maori-New Zealander.

^c^Other (self-described): Afrikaans South African, Burmese, and Iranian.

### Usage Data

Table 3 presents the MYLO usage data. Participants’ usage data were collected in the MYLO app while their survey responses were collected anonymously via an external host (Qualtrics). Therefore, it was not possible to link participant outcomes to their MYLO usage in this phase, and the usage data reported is for all conversations had by all participants who downloaded MYLO and had at least one conversation during the trial period (n=22).

**Table 3:** MYLO (Manage Your Life Online) Usage data divided by number of messages, conversations, and users.

		Values
Number of users, n		22
Total conversations, n		90
Conversations per user, mean		4.1
**Number of messages from each user per conversation**		
	Mean (SD)	11.54 (11.10)
	Median (range)	8.5 (1-61)
	<5 messages	25 (28)
	5-10 messages	26 (29)
	11-20 messages	29 (32)
	≥21 messages	10 (11)
**Conversation rating, n (%)**		
	Helpful	14 (16)
	Neither	16 (18)
	Unhelpful	37 (41)
	Unrated	23 (26)

### Conversation Decryption and Consent Acceptability

All 24 participants who completed the baseline survey were sent an information sheet and consent form via email to allow the research team to decrypt and analyze the conversations they had with MYLO during the project. Of the 24 participants, four consented to their conversations being analyzed, one responded saying they did not want their conversations decrypted and analyzed, and 18 participants did not respond to the email. One participant let the research team know they would complete their consent form via email, but then did not contact the team again. From these participants, we were able to collect 14 conversations, which will be analyzed and presented in a future paper.

### Adverse Events

In total, 8 participants completed the adverse events survey. All items were scored 0 (Not at all), 1 (Very little), 2 (A little), 3 (Quite a lot), or 4 (Very much). “Not Applicable” responses were not scored or included in calculations. A total of 25 items in the survey describe adverse events, and an average score of each was calculated for each participant was calculated. These scores ranged from 0.04 to 2.48 (mean 0.81, SD 0.78). Only 2 participants achieved a mean of over 1 on the scale. A total of 9 items had mean scores between 1 and 2. Three of these items related to the time and effort required to participate. One item scored 2.38, and that was “Taking part hasn't helped me with my problems.” Item 26 is a positive item and asks participants’ agreement with the statement “My problems have improved to the point whereby I no longer feel I need help,” and this had a mean score of 2. Participants were also able to provide qualitative feedback on the adverse events survey, and 4 participants chose to do so. Three participants left positive comments about MYLO, one of whom chose to explain their responses on the adverse event survey:

I had a very easy time figuring out how MYLO worked, but midway had a bout of depression that was unrelated to using MYLO, which made it very difficult for me to find the motivation to use the app.

Doing the MYLO trial helped me have a better understanding of mental health and how it can affect you and the people around you. It definitely made me have a larger understanding of how much impact I can have on all the daily activities you do and your thoughts and mindset. I think that MYLO was a very well-created system, and I hope that our community can also enjoy and learn from it.

I think the MYLO experience was overall good. I can't wait to see the following phases of MYLO as I can see this AI has the basic structure to become something that is going to be very helpful towards a large population of people.

One participant left a negative response:

During times of mild anxiety/dissociation/depression, etc., I tried to use MYLO to help support me instead of my usual support system. MYLO was very unhelpful and made the problems worse due to its inability to provide any advice, practical help, or even have a proper conversation about the topic of mental health issues. Because of this I found that I needed to use other support systems to not only help with the original problem but counterbalance the lack of support that I felt from MYLO.

### Wellbeing Outcomes

Table 4 presents the mean scores for each self-report questionnaire at each time point as well as the change from baseline to week 4. Table 5 presents how many participants achieved reliable improvement or deterioration from baseline to posttesting for all outcomes. We found a large effect size for problem-related distress and a mean change of 3.77 from baseline to week 4 (Cohen *d*=–1.07). Anxiety scores showed a mean reduction of 2.08 (Cohen *d*=–0.41), indicating a small-to-moderate reduction from baseline to week 4. A medium effect size was found for psychiatric impairment, with the mean score posttesting dropping below the case threshold (ie, clinical threshold for psychiatric impairment) of 3 [30] to 2.46 (Cohen *d*=0.60). Of the 13 participants who completed both the baseline and posttesting (week 4), 11 individuals scored 3 or more at baseline, and 6 scored 3 or more posttesting (refer to Multimedia Appendix 2). The reliable change scores show that, on the measures of problem-related distress, 6 participants achieved a reliable improvement, and none had a reliable deterioration. For anxiety, 2 participants achieved a reliable improvement, and none had a reliable deterioration. For psychiatric impairment, 6 achieved a reliable improvement, and one had a reliable deterioration.

**Table 4:** Mean scores on clinical outcomes at baseline, week 1, week 2, week 3, and week 4.

	Baseline (n=24), mean (SD)	Week 1 (n=17), mean (SD)	Week 2 (n=14), mean (SD)	Week 3 (n=12), mean (SD)	Week 4 (n=13), mean (SD)	Change^a^, mean (SD)	95% CI	*d^b^*
General health	0.56 (0.25)	0.69 (0.19)	0.71 (0.17)	0.65 (0.36)	0.71 (0.22)	0.04 (0.09)	–0.02 to 0.09	0.44
Depression	12.54 (5.45)	10.35 (4.18)	8.71 (4.34)	9.00 (5.31)	9.23 (5.89)	–1.85 (3.96)	–4.24 to 0.54	–0.47
Anxiety	9.58 (4.68)	8.82 (4.67)	8.00 (3.72)	8.58 (4.32)	7.08 (4.03)	–2.08 (5.02)	–5.11 to 0.96	–0.41
Psychiatric impairment	5.63 (2.58)	3.06 (2.73)	2.57 (2.85)	2.92 (2.81)	2.46 (2.96)	–2.31 (3.86)	–4.64 to 0.03	–0.60
Goal conflict reorganization	66.81 (13.12)	63.53 (10.40)	62.21 (13.38)	64.08 (15.20)	64.92 (15.06)	–1.00 (16.50)	–10.97 to 8.97	–0.06
Self-efficacy	28.71 (4.75)	28.53 (4.16)	28.93 (5.37)	28.17 (4.39)	29.46 (4.39)	0.31 (4.13)	–2.19 to 2.80	0.07
Problem-related distress	13.96 (2.71)	10.53 (2.21)	10.14 (2.96)	10.75 (2.99)	9.00 (3.46)	–3.77 (3.52)	–5.89 to –1.65	–1.07

^a^The change column presents the mean change between baseline and week 4; only scores of those who completed the week 4 survey are included (n=13).

^b^Cohen *d* was calculated using Jamovi for each outcome posttesting (end of week 4) relative to baseline [36].

**Table 5:** Participants’ change scores from baseline to posttesting for all target outcomes.

	Cronbach α	Reliable Change Index [33]	Participants’ scores												
			1	2	3	4	5	6	7	8	9	10	11	12	13
General health	0.82	0.26	–0.010	0.111	0.053	0.038	–0.126	0.000	0.029	–0.037	–0.005	0.165	0.251	0.014	0.054
Depression	0.86	5.78	–1	–4	–7^a^	–1	3	–5	2	6^b^	–1	–1	–8^a^	–3	–4
Anxiety	0.85	5.72	1	–2	–4	–2	1	–4	4	3	–1	–8^a^	–15^a^	1	–1
Psychiatric impairment	0.78	3.44	–2	–1	–4^a^	3	4^b^	–7^a^	3	0	–5^a^	–5^a^	–7^a^	–3	–6^a^
Goal conflict reorganization	0.81	13.90	–14^b^	17^a^	30^a^	11	–33^b^	–5	–5	5	–12	2	6	5	–20^b^
Self-efficacy	0.86	4.57	–1	1	7^a^	–1	–8^b^	–2	–2	5^a^	1	6^a^	2	0	–4
Problem-related distress	0.72	3.62	–4^a^	0	–12^a^	1	–7^a^	–1	–2	–2	–3	–2	–6^a^	–7^a^	–4^a^

^a^Denotes participant who achieved reliable improvement.

^b^Denotes participant who achieved reliable deterioration.

We found no effect on goal conflict reorganization with a mean change of 1.00 from baseline to week 4 (Cohen *d*=–0.06). The reliable change scores show that 2 participants achieved a reliable improvement in their ability to reorganize goal conflicts, and 3 reliably deteriorated.

Participants showed a small-to-moderate effect size improvement in general health between baseline and posttesting (Cohen *d*=0.44, mean change=0.04). No participants experienced a reliable change in general health. We found a small-to-moderate effect size on participants’ depression scores between baseline and posttesting (Cohen *d*=0.47, mean change=1.85). We found no effect on self-efficacy (Cohen *d*=0.07, mean change=–0.31). The reliable change scores show that no participants experienced a reliable change in their general health, 2 participants achieved reliable improvements in their depression scores, one participant reliably deteriorated, 3 participants achieved reliable improvements in their self-efficacy scores, and one participant reliably deteriorated between baseline and posttesting.

### User Experience

In the current and previous case-series [22], participants rated MYLO’s usability using the System Usability Scale [33] every week during the testing phase (over 4 weeks and 2 weeks, respectively). In the previous case series, MYLO’s System Usability Scale ratings ranged from 37.5-97.5, with a mean rating of 73.57 (SD 16.02). In the current case series, the ratings ranged from 40 – 97.5, with a mean rating of 70.67 (SD 14.59). For both case-series, the mean ratings indicate MYLO is “acceptable” to most users, and scores were above average [37]. This suggests users’ perceptions of MYLO’s usability did not change between the 2 case series, and the system is performing similarly to the previous case series.

### Accessibility of MYLO Interface

These results were echoed in the qualitative data, as many of the participants were generally positive about MYLO “I think it's rad. I think the idea is great” (16-year-old). All the participants described the MYLO interface as easy to use “overall for me, I found it really easy to use” (17-year-old). Many participants liked the accessibility of being able to talk about their problems anywhere “I could just click on the app when I wanted to use it, it would just start right away...it was very convenient” (17-year-old). Please note that quotes throughout have been edited lightly for clarity.

Some participants described times where they would prefer to use MYLO instead of talking to a person, “I found it useful in that respect…instead of feeling like I had to…sit down and talk to someone about it” (23-year-old). These participants were in 2 groups: those who would use MYLO when the people they would usually talk to were not available,

I do a lot of thinking in the nighttime…around those times when I'd be sorting problems out. And it's good to have MYLO...especially at those times.

16-year-old

And those who were reluctant to talk to people about their problems,

it was those points that I sort of reached for MYLO. I don't tend to be someone that usually reaches out and talks to someone about this stuff. I'm more private about it.

17-year-old

One participant felt they were able to share more authentically than if they had been talking to a person,

Having someone right there in front of you and looking you dead in the eyes, that's really confrontational. For me personally, I'll get nervous...now I don't feel comfortable talking about this. So it [MYLO] definitely felt comfortable. Just to be able to say it as it was.

23-year-old

Some participants felt the familiar chat format was helpful to support problem-processing,

I prefer that [texting], I wouldn't mind talking on the phone, but I think it's nicer to be able to [text]. When I'm typing, I can process it better in my mind and it helps me come to terms with what I'm upset about.

17-year-old

and made them feel comfortable,

I like the chat format setting because, I guess it almost feels like you're chatting with a friend in a way, because it's like a text message. So that provides almost a sense of comfort.

17-year-old

### Satisfaction With Therapy Sessions

We compared participants’ satisfaction with MYLO’s therapeutic conversations in the current case series to the previous case series. Table 6 presents the mean therapy satisfaction scores measured by the Session Impact Scale [32] across all weeks (excluding baseline, where the scale was not included) for all participants. We found a large difference in (standardized) effect sizes between the previous and current case series on the hindering impact scale, with current case-series participants rating MYLO higher on the subscale (indicating a larger hindering impact) than the previous case-series' participants. We also found a moderate difference in (standardized) effect sizes between the previous and current case series on unwanted thoughts. The higher rating in the current case-series indicates that participants experienced more unwanted thoughts than the previous case-series’ participants.

**Table 6:** Session impact subscale scores for Manage Your Life Online (MYLO) in the current case-series and the previous case-series [22].

Session impact subscale^a^	Current case-series (n^b^=17)	Previous case-series (n=11)	Mean difference (95% CI)^c^	Cohen *d*
Understanding, mean (SD)	2.11 (0.61)	2.36 (0.99)	0.26 (–0.45 to 0.97)	0.33
Problem-solving, mean (SD)	1.91 (0.82)	2.09 (1.04)	0.18 (–0.60 to 0.96)	0.20
Relationship, mean (SD)	2.02 (0.83)	2.22 (0.92)	0.20 (–0.52 to 0.91)	0.23
Hindering, mean (SD)	2.49 (0.87)	1.76 (0.52)	–0.74 (–1.28 to –0.20)	–0.98
Unwanted thoughts, mean (SD)	1.84 (0.70)	1.50 (0.50)	–0.34 (–0.81 to 0.12)	–0.54

^a^Session impact subscale score: 1=not at all, 2=slightly, 3=somewhat, 4=very much, and 5=very much.

^b^Number of responses across all weeks of each case-series.

^c^The mean and SD were calculated by first calculating the mean for each participant in each case-series for each subscale, and the table then displays the mean of these means. The mean difference is the mean from the previous case-series minus the mean from the current case series, and so represents the reduction in mean session impact score from the previous to the current case-series. The SD to perform the standardization for Cohen *d* is the pooled SD across the two case series.

### Helpfulness of MYLO Conversations

Some participants found the conversations they had with MYLO useful, and that MYLO achieved its intended goal, “Sometimes it genuinely did make me reflect on my thoughts and stuff like that. Like with those questions” (16-year-old),

You know one of the defining characteristics for rumination, it's unproductive and persistent. So, the biggest thing for me about MYLO was that it felt productive because I think it helped me to think about things in a different way.

23-year-old

One participant felt at times that MYLO could do a better job of helping the participant than a person,

And I almost felt like it's therapeutic…I can just type it [a problem] out and then MYLO would just pick out all the important bits for me. And I don't have to focus. Whereas I feel like sometimes when you… [are] talking to people about it [a problem] …you sometimes [have to] give them like all the context, and they'll get confused in the small details.

23-year-old

### Difficulties With MYLO Conversations

Despite some positive feedback, the Session Impact Scale scores suggest that participants experienced hindering effects when using MYLO. Participants described 4 main problems: MYLO’s questions were generic, MYLO was repetitive, MYLO had a limited purpose, and MYLO was not “as good” as some freely available generative AI chatbots.

#### Repetitive

Like the previous case series, the largest reported problem participants had with MYLO was repetition or looping. Although MYLO is programmed not to repeat the same question for at least 20 exchanges, participants felt questions were sometimes too like each other, “It just seems to be quite repetitive” (16-year-old), or that MYLO asked questions about a topic they had already discussed, “Like sometimes I felt like I was going in circles. Like I'd say something. Then it'll give me a question and I'll be like, I just told that” (17-year-old).

#### Generic

Participants also felt that sometimes MYLO asked generic questions, “The only thing that I sort of felt was on the downside is that sometimes, especially if you're talking about a problem that's quite specific, it would only give general answers” (17-year-old). One participant was conflicted as they sometimes found the similar or general questions useful, but at other times they caused frustration, “[MYLO] asking you to write more, sometimes was a bit frustrating when I didn't have anything to add. Sometimes it did help me to expand on what I was thinking and realize that maybe it's more complicated” (17-year-old).

When either of these issues (ie, repetitive or generic questioning) occur in a conversation they disrupt the flow of conversation and cause the participant to disengage, “It sort of jolted me backwards out of the conversation. I didn't expect [that response], that's not a response I would ever expect from a person that I was talking to” (19-year-old). “If you'd kind of forgotten for a bit, you were reminded that this is just a computer” (23-year-old)**.**

#### Limited Purpose

Participants also felt that MYLO had a limited purpose and functionality at times. MYLO is programmed to only ask curious questions about a problem, and therefore does not engage in other forms of dialogue. Some participants wanted MYLO to engage in more casual conversation with them and suggested MYLO ask questions about their everyday life as well, “for people who have like a good day…or even just a bit lonely [but] you're still OK…I just want to have a bit of conversation” (17-year-old). This could improve MYLO's scores on the session impact scale, as some participants did not currently feel understood by MYLO: “It doesn't feel like it really knows you” (17-year-old).

#### Comparisons to Generative AI Chatbots

A novel critique that participants described (6 out of 13), which did not appear in the previous case-series [22], was how MYLO compared to freely available generative AI chatbots (such as ChatGPT). Participants had experience with generative AI chatbots, “ChatGPT started blowing up this year as well. The top 2 examples [for] me personally I would take #1 being Snapchat and second being ChatGPT” (16-year-old). Although not all participants were as positive about generative AI or digital therapies,

Knowing that it's a computer sometimes I don't take it super seriously or don't think that it's gonna be as helpful and that in itself can be a bit of a barrier. I'm not maybe trying or engaging with it as well as I could just because I'm not expecting it to be helpful.

17-year-old

Many had expectations that MYLO would look, “I guess something like the interfaces of AI chatbots, like for example like ChatGPT. [Were] what I was kind of expecting…it kind of like subverted my expectations” [16 years old], and behave like other generative AIs (ie, be able to answer user questions and have considerable natural language processing skills). Some were disappointed when these expectations were not met,

I've used ChatGPT a bit…that [ChatGPT] kind of framed it for me that I was expecting [a similar experience] …but that's where I got to a bit of a dead end with it [MYLO]. I wanted to ask it things like give me…some time management strategies.

23-year-old

In the last case series, most participants would recommend MYLO to their friends, whereas the current participants appear to have a higher expectation for natural language programs, given the inception of ChatGPT and other generative AIs.

Definitely could be something I would recommend to other people, but maybe in this stage kind of what he (23-year-old) was saying before when you compare it to things like ChatGPT, which obviously have so much more money and like people behind it, it does kind of fall short.

17-year-old

Participants had some specific issues and recommendations informed by their knowledge and experience of generative AI chatbots. For example, ChatGPT has a context window that allows it to “remember” what a user types within each conversation [38]. MYLO, on the other hand, uses the immediate user response to generate its next question rather than all responses within a conversation, aside from rules included to avoid repetition of the same question within a certain number of messages. This is by design, so that MYLO is better able to emulate an method of levels therapist and respond to current statements made by the user. Participants, however, wanted MYLO to recall everything they said, in line with their perception of how ChatGPT operates,

I think that would be really, really useful, because when you look at ChatGPT like I've definitely used it and had asked it a question and then asked a question that was related to what it said and the previous question like following on and it's being able to respond and adjust. So I think that sort of capability in MYLO would definitely, really improve the conversation and would also probably really improve the chance of me recommending it.

19-year-old

Other participants enjoyed the free text responses they got from generative AI chatbots and the ability to have a general conversation with them, compared to MYLO’s preauthored and structured responses.

I feel like MYLO doesn't really do that because of the fact that it doesn't have the free speech. As I mentioned before, like for example ChatGPT, …you can actually have a conversation with them, for example, the Snapchat AI.

16-year-old

Despite these comments, participants did recognize the dangers of generative AI, “it's a bit more risky, like you have to make sure where the information is drawing from” (17-year-old), and the therapeutic benefit of MYLO’s style of conversation,

It's good that MYLO was just always asking me questions. In any sort of therapy setting, I guess it's better that the client would do more of the talking and the therapist would do more listening and asking questions.

23-year-old

## Discussion

### Principal Findings

We aimed to build on the acceptability and feasibility analysis within a previous shorter case-series [22] by extending the time that users had access to MYLO, and by increasing the types of data collected; specifically, we added an adverse events survey and the potential for participants to share the conversations they had with MYLO with the research team. The addition of the adverse events survey allowed us to further assess the safety of MYLO as an intervention, and the results suggest that no adverse events were attributed to MYLO, although one individual found its lack of advice made them feel worse and caused them to seek advice from their usual supports. Although MYLO’s instructions state that MYLO only asks questions and will not give advice, some users might not read the instructions. To avoid other users experiencing this problem in the future, MYLO’s purpose and functionality could be made clearer and unavoidable upon sign-in or when opening a conversation.

Only a small number of participants (4 out of 24) consented to the research team decrypting and analyzing their conversations in this case series. It is possible that participants are uncomfortable sharing their conversations with MYLO due to the sensitive nature of the conversations. It is also possible that the method of informing and collecting consent contributed to this low participation. In future studies, we aim to address both issues by implementing a method for participants to provide consent within the MYLO interface for each conversation they would be willing to have analyzed. This method will increase participants’ autonomy over their data and provide an easy and accessible way for participants to provide consent.

To evaluate MYLO’s ability to support the well-being of young people, we also assessed the effect size of the change over time in key outcome variables.

Participants’ problem-related distress, anxiety, and psychiatric impairment showed substantial effect sizes for improvement after testing MYLO for 4 weeks. These findings were in the same direction as the previous case-series [22].

No change was found in participants’ ability to engage in goal conflict reorganization. Inspection of the reliable change scores for all participants showed that 2 participants achieved reliable improvement, while 3 reliably deteriorated. It is not clear why these results were different from the previous case-series [22], which showed a medium effect size increase in goal conflict reorganization. Goal conflict reorganization is the proposed mechanism of change for method of levels and MYLO, by increasing clients’ and users’ ability to resolve the conflicts they experience, their problem-related distress should improve, and subsequently their depression and anxiety symptoms should reduce [21]. Several method of levels studies have demonstrated this increase in ROC scores [39-41]. Further research is needed to establish the impact of using MYLO on goal conflict resolution with a fully powered sample. If MYLO provides the opportunity for goal conflict reorganization to be implemented via its interface for problem expression and curious questioning, then we would expect greater evidence of goal conflict awareness in the conversations to mediate its benefits, as found in an earlier study [21]. As it stands, the current study challenges the conceptual credibility of this mechanism, in contrast to earlier studies. Tentatively, it is possible that participants’ expectations for near-human conversational ability based on generative AI—which was not available during earlier research on MYLO—reduced their commitment to engaging in depth with MYLO regarding their goal conflicts. We discuss this in more detail later.

We found an effect on depression scores, and the reliable change score of individuals suggests some participants experienced an improvement in their self-efficacy scores. However, more research in a fully powered randomized trial is needed to validate these findings. The null findings related to general health could be due to the items in the Short Form-6D version 2, which focus on 6 domains of health, including mental health. MYLO’s focus on mental health and well-being limits its ability to improve the other domains in the Short Form-6D version 2, unless a user is seeking to explore a problem related to one of the other domains.

Regarding the user experience of MYLO, it scored similarly on the session impact scale to the previous case series, except for achieving a higher score on the hindering impacts subscale. As the system usability scale scores were similar, suggesting participants found MYLO similarly acceptable to the participants in the last case-series, we examined the qualitative data to find a possible explanation for this difference. Much of the positive qualitative feedback was similar to the previous case-series and other mental health chatbots [5,6]; participants praised the accessibility of the interface. Participants also described incidents where MYLO had helped them to achieve a new perspective on their problems. Participants also experienced similar difficulties regarding the repetition of questions and instances when MYLO did not understand them. These are difficulties that many chatbots, especially logic or rule-based chatbots, experience [9]. In our study, only 16% of conversations were rated as helpful, which is clearly nonoptimal. However, there appears to be no data from other conversational agents for comparison.

One difference that emerged was comparisons between MYLO and widely available generative AI models such as ChatGPT and Gemini. The widespread availability of these chatbots appears to have altered users’ expectations of what interacting with a chatbot is like. Our previous participants had not used these models, as they were not yet available, and while they experienced similar issues and scored MYLO similarly on all other session impact scale subscales (ie, understanding, problem-solving, and relationship), they were much more likely to recommend MYLO in its current form. In contrast, the current participants were less likely to recommend MYLO, despite similar reductions in problem-related distress and other mental health symptoms. Specifically, our users pointed to the natural language processing and generative AI chatbot’s ability to adapt and perform many tasks (from writing computer code to answering complex questions on many topics) as superior to MYLO’s specific question-answer structure.

Many mental health chatbots lack the functionality of large generative AI models for many reasons (eg, funding), but often by design. For example, as one participant noted, it is often beneficial for the client to do most of the talking during a therapeutic conversation, whereas current generative AI models produce large amounts of text in response to minimal user input. Another drawback from generative AI recognized by our participants was the lack of transparency regarding where information or advice came from, whereas MYLO and other mental health chatbots generate text previously authored by mental health professionals, or generate text constrained by very specific rules created by mental health professionals [42]. Despite this, as technology advances, these interventions might no longer meet users’ standards for how naturalistic a digital conversation should be. This could exacerbate the already difficult issue of retention in digital interventions and ultimately limit the effectiveness of MYLO and other mental health chatbots.

One potential explanation for the contrast we found is that rule-based chatbots and generative AI chatbots occupy opposite ends of the “uncanny valley” [43]. The uncanny valley was originally developed to describe a midground between simulations of humans that are clearly experienced as a computer, and those simulations that are almost indistinguishable from humans. When users know they are conversing with a computer, they can nonetheless use its benefits without the concern of sharing their thoughts and feelings with another human. This is thought to be the main reason for what has come to be known as the Eliza effect, identified early on in chatbot development [44], and is likely to have been operating in early versions of MYLO. Yet as computer simulations occupy the midground between a computer and a human, the appeal of the simulation is found to be substantially worse than either extreme and is associated with a feeling of eeriness and detachment from the agent. A recent systematic review indicates that this effect is present in chatbots, with users reverting to either side of the uncanny valley—more simplistic chatbots, or to humans—when their chatbot interaction is near-human [45]. It is possible that the improvements of MYLO, in the context of recent human-like text-based conversations with generative AI, led perceptions of MYLO into the uncanny valley. Indeed, it has been shown that a text-based chatbot programmed to create the uncanny valley effect is experienced as less likable and intelligent than a chatbot programmed to converse exactly like a human [46]. Our findings may therefore reflect the challenge of the uncanny valley regarding the attempt to improve rule-based chatbots through simply integrating generative AI capable of natural conversation; codesign and user testing will be needed to mitigate the issues.

### Future Directions

The concerns regarding generative AI have been expressed more widely by researchers [47-50]. These include the significant cost and environmental impact of maintaining open-access models, which may become prohibitive for widespread applications as costs shift to consumers [51]. In addition, maintaining conversational context is resource-intensive, a limitation noted across systems like ChatGPT and MYLO, though OpenAI’s retention of previous discussions illustrates attempts to address this [52]. Generative AI struggles with handling ambiguity, often generating poor responses to unclear inputs, and lacks true reasoning capabilities, relying instead on probabilistic predictions based on training data [52]. Emotional intelligence is another limitation, as these systems, including MYLO, cannot currently detect or adapt to users’ emotional states. Furthermore, ethical concerns arise from biases in training datasets, particularly in sensitive areas like mental health [49,50,53]. Unlike commercial Generative AI tools, MYLO’s design potentially offers a more controlled and transparent approach to mitigating these risks, and future research could make a direct comparison of benefits and risks between MYLO and a purely generative AI mental health chatbot.

More critically, MYLO’s focus exclusively on curious problem exploration and noticing thoughts and feelings has 2 additional advantages. It guards against the issues with advice, misinformation, and sycophancy in generative AI, and supports the intrinsic capacities for users to solve their own problems owing to MYLO’s scientific foundations in perceptual control theory [20]. From the perspective of perceptual control theory, longstanding distress emerges from unresolved goal conflicts that require sustained exploration to resolve through a process known as reorganization. Conversely, it is proposed that the advice and information may support the pursuit of the conflicting goals, actually resulting in greater distress [54].

Currently, the logic-based system behind MYLO uses human-selected therapeutic questions about specific themes, and it prioritizes certain questions over others based on the ranking of topics. For example, terms indicating goal conflict, such as “dilemma” or “in two minds,” are ranked higher than terms about activities, such as “going to the gym.” In addition, questions that are consistently rated by past users as unhelpful are less likely to be selected. These elements will remain, yet they will be supplemented by large language models to address some of the recognized issues. First, we have convened a new, diverse consumer advisory group to make recommendations, give feedback on planned improvements, and carry out user testing. This will proceed in iterative steps to ensure that each phase of improvement enhances the user’s experience of MYLO. We are scoping for a natural language processor that is secure and energy efficient to hybridize with MYLO. Its 2 main objectives will be to ensure that themes are more readily and accurately recognized, and to ensure that MYLO selects a response that is appropriate and makes sense to the user. We expect that this hybrid version will improve the user’s sense of being understood, maintain their engagement, and ensure that their conversations are long enough to allow for detailed problem exploration, promoting the reorganization of goal conflict as proposed by perceptual control theory [20]. Whilst the exact specifications of the hybridization are unclear at this stage, we plan to retain the existing rule-based selection of prespecified theory-driven questions, to prevent it from confusing the user with a shift in therapeutic principles [17], and to limit the recognized risks of computer-generated text in the arena of mental health [55].

### Limitations

We used a case-series design to evaluate MYLO and assess the acceptability of new data collection methods. The lack of a control group, small sample size, and high dropout rates, both in terms of maintaining engagement with MYLO and providing outcome data, suggest that our findings are indicative of a selective group that is not methodologically robust. The findings of this study will be used to inform the design of a randomized controlled trial that compares MYLO users to a waitlist group to provide a robust and valid assessment of MYLO’s efficacy. Second, we also experienced high levels of dropout between baseline and the first week of testing when participants were required to download and log in to MYLO, which may have biased the final sample. This step had been added to this case series. It was not necessary in the previous MYLO projects [22] where participants were able to use MYLO without an account through a weblink. The current process requires the participant to inform the lead researcher that they have completed the baseline survey; the lead researcher then creates an account for the user and emails or texts them their login details, as well as instructions on how to download and use MYLO. This process was a necessity for this stage, given the authentication platform being used (Auth0) and MYLO's current stage of development. However, previous consumer panel members have indicated a preference for password-less access, stating that passwords and usernames are a barrier for some young people [22]. We therefore aim to address this issue in the next research and development phase by allowing users to create their own accounts and let them use their saved passwords, biometrics or social media log ins to authenticate their identity; we will also link usage data to individual participants whilst preserving their anonymity so that the potential dose-response relationship between usage and outcome can be analyzed to address this analytic limitation within the current study. Ultimately, the goal is to create a smartphone application version of MYLO that can be placed on all available application stores. Doing so will greatly increase MYLO’s accessibility and availability to a broader sample of users.

The number of participants in the qualitative analysis was not guided by data saturation or thematic saturation. We are aware that this contradicts good qualitative practice and may have introduced a selection bias, and so would not be considered generally representative of potential users of MYLO. We also note that our framing of experiences with MYLO after the widespread use of generative-AI chatbots was opportunistic in the absence of similar studies, yet a comparative or crossover design would have been ideal.

### Conclusions

This study aimed to extend our previous work on MYLO by providing the application for a longer period of time, 4 weeks, and examining its impact on MYLO users’ well-being. We also assessed the feasibility of changes to the trial methodology, that is, adding the option for participants to consent to decryption and analysis of their conversations before conducting a full-scale randomized controlled trial. All changes will be retained moving forward, but we are hoping to integrate consent to decrypt conversations into the application interface to increase the number of participants opting in and to allow participants to opt in for individual conversations. Regarding the quantitative results, the direction of the pre-post effects on problem-related distress, anxiety, and psychiatric impairment was the same as previous research on MYLO. However, the lack of change in participants’ goal conflict reorganization and the mixed findings on self-efficacy and general health highlight the need for further investigation with a fully powered randomized controlled trial to better understand the intervention’s impact on participants’ well-being and mental health, as well as the mechanisms driving these changes.

The study also revealed key insights into the user experience of mental health conversational agents, particularly in comparison to widely available generative AI models like ChatGPT and Gemini. While MYLO scored similarly on user experience and usability metrics, the changing expectations of users in response to advances in AI technologies suggest that integrating more sophisticated natural language processing features might enhance its usability, rapport-building, and therapeutic outcomes. Further, addressing technological barriers, such as streamlining user authentication and developing a smartphone application, will enhance MYLO’s accessibility and engagement. Future iterations of rule-based chatbots, incorporating co-designed improvements and hybrid approaches leveraging large language models, hold the potential to better meet users' evolving expectations and improve retention in digital mental health interventions.

## Appendix

Multimedia Appendix 1 Topic Guide for the Focus Groups and Interviews.

Multimedia Appendix 2 Spreadsheet presenting the baseline and 4 week (post-testing) scores on all clinical outcomes for completers.

## Abbreviations

AI: artificial intelligence
MYLO: Manage Your Life Online
PHQ-9: Patient Health Questionnaire-9
